# Promotion of Hair Growth by Conditioned Medium from Extracellular Matrix/Stromal Vascular Fraction Gel in C57BL/6 Mice

**DOI:** 10.1155/2020/9054514

**Published:** 2020-06-13

**Authors:** Shune Xiao, Yurong Deng, Xiaojin Mo, Zhiyuan Liu, Dali Wang, Chengliang Deng, Zairong Wei

**Affiliations:** Department of Plastic Surgery, Affiliated Hospital of Zunyi Medical University, Zunyi, Guizhou, China

## Abstract

Adipose-derived stem cell- (ADSC-) based regenerative medicine has expanded to include the treatment of hair loss. However, stem cell therapy remains a relatively recent technique, and reports of its use for treating alopecia are rare. ADSCs exert biological functions via the paracrine actions of various growth factors and cytokines. Conditioned medium from ADSCs (ADSCs-CM) is a cell-free suspension rich in growth factors and cytokines that has demonstrated a significant role in stimulating hair growth, with encouraging outcomes in terms of hair regeneration and hair growth. Extracellular matrix/stromal vascular fraction gel (ECM/SVF-gel) is an ADSC- and adipose native extracellular matrix-enriched product for cytotherapy. In this study, we compared the effects of CM from ECM/SVF-gel (ECM/SVF-CM) and from stem cells (SVF-CM) on hair growth in mice. ECM/SVF-CM stimulated hair growth more than SVF-CM, through promoting the proliferation of dermal papilla cells and cells in the bulge, neovascularization, and anagen induction. ECM/SVF-CM might, thus, provide an effective and improved strategy for promoting hair growth. These data provide a theoretical foundation for the clinical administration of ECM/SVF-CM for the treatment of hair loss.

## 1. Introduction

Hair loss is one of the most common complaints presenting to dermatologists and plastic surgeons and is usually associated with a major psychological impact on the affected patient. Androgenic alopecia is a major cause of hair loss, with an estimated 70% of men and 40% of women affected by androgenic alopecia and hair loss at some point in their lives [1]. Conservative treatments for alopecia include the use of drugs, such as finasteride and minoxidil, and surgical hair transplantation [2]. However, these treatments are associated with their own adverse reactions and are ineffective in some patients.

Alternative safe and effective therapeutic approaches are therefore required. Using the patient's own stem cells to regenerate hair growth is a promising alternative therapeutic strategy [3]. Adipose-derived stem cells (ADSCs) are mesenchymal stem cells within the stromal vascular fraction (SVF) of subcutaneous adipose tissue, which have been extensively researched and have been applied in tissue engineering and regenerative medicine [4, 5]. Furthermore, the field of ADSC-based regenerative medicine has now expanded to include the treatment of hair loss. Cellular therapy with human autologous SVF ADSCs was recently reported for alopecia areata, with encouraging outcomes including increased hair density and thickness and decreased pull-test results [6]. However, stem cell therapy is relatively novel and subject to regulatory surveillance, and reports of its use for the treatment of alopecia are rare. ADSCs exert their biological functions via the paracrine actions of various growth factors and cytokines [7]. Innovative approaches to hair regeneration involve the production of conditioned medium from ADSCs (ADSCs-CM) and use of this cell-free suspension enriched with nutritious growth factors and cytokines secreted by ADSCs [8]. ADSCs-CM has been reported to be rich in growth factors and cytokines, including vascular endothelial growth factor (VEGF), basic fibroblast growth factor (bFGF), platelet-derived growth factor (PDGF), hepatocyte growth factor (HGF), keratinocyte growth factor (KGF), and insulin-like growth factor I (IGF-I) [8]. These growth factors and cytokines potentially modulate the host environment and thereby play significant roles in hair growth [9]. Previous studies found that human hair regrowth and density increased significantly after the application of ADSCs-CM [10–13].

The generation of specific CM rich in growth factors/cytokines to support hair regeneration and growth is the essence of therapeutic strategies, and several approaches can be used to increase the levels of ADSC growth factors/cytokines. A low dose of UVB radiation was shown to upregulate the secretion of ADSC-derived growth factors, and treatment with UVB-irradiated ADSCs-CM improved hair regeneration and modulated the hair cycle in mice [14]. Hypoxia also enhanced the paracrine effects of ADSCs [15]. Other strategies to increase the secretion of growth factors/cytokines include growing the ADSCs in 3D or spheroid cultures or culturing the cells in laminin- or Matrigel-coated culture vessels [16–18]. Lu et al. described an injectable adipose tissue-derived stem cell and adipose native ECM enrichment product (ECM/SVF-gel) [19] generated from the Coleman adipose tissue using a pure mechanical process, which eliminated adipocytes while preserving ADSCs/SVF [19]. They recently demonstrated that ECM/SVF-gel exhibited greater effects on wound healing compared with SVF suspension, mainly as a result of the high concentration of growth factors secreted by the ECM/SVF-gel [20]. Furthermore, CM from ECM/SVF-gel was rich in growth factors and improved wound healing compared with conventional SVF-CM [21].

We therefore hypothesized that CM from ECM/SVF-gel culture might be an attractive source of multiple growth factors for hair growth. To test this hypothesis, we prepared CM from ECM/SVF-gel (ECM/SVF-CM) and SVF cultures (SVF-CM) and compared the effects of these CMs on hair growth. We studied the effects of the CMs on the proliferation of human dermal papilla cells (DPCs) *in vitro* by the Cell Counting Kit-8 (CCK8) assay and investigated their effects on hair growth in C57BL/6 mice *in vivo*. Furthermore, we identified growth factors present in the CMs potentially responsible for this stimulatory effect.

## 2. Materials and Methods

### 2.1. Animals and Ethical Approval

Wild-type, 7-week-old C57BL/6 mice were provided by the Animal Experimental Center of the Army Military Medical University (Chongqing, China). Human adipose tissue was obtained during fat-grafting procedures, and normal human scalp hair follicles were obtained during hair transplantation procedures, after obtaining written informed consent. All protocols were approved by the Ethics Committee of the Affiliated Hospital of Zunyi Medical University. All animal experiments were approved by the Institutional Animal Care and Use Committee of the Affiliated Hospital of Zunyi Medical University and conducted according to the guidelines of the National Health and Medical Research Council (China).

### 2.2. Preparation of CMs from ECM/SVF-Gel and SVF

**Figure 1:**
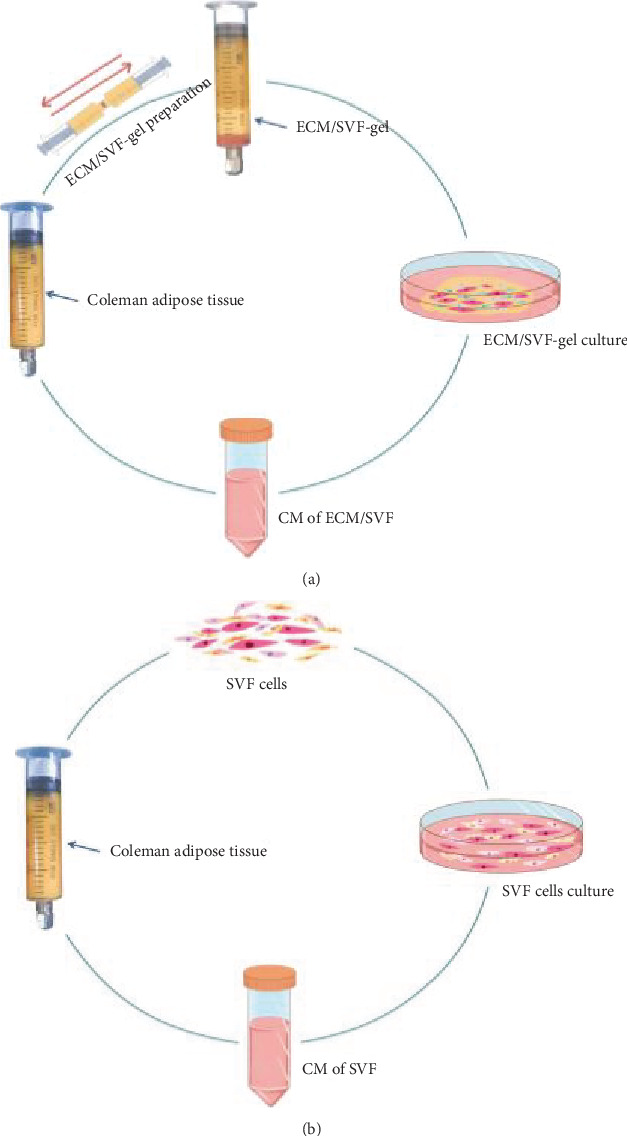
Schematic diagram of the preparation of ECM/SVF-CM (a) and SVF-CM (b). ECM: extracellular matrix; SVF: stromal vascular fraction; CM: conditioned medium.

ECM/SVF-gel and SVF suspension were prepared as described previously [19]. Briefly, the Coleman adipose tissue was mechanically emulsified by shifting between two 10 mL syringes connected by a Luer-Lok connector with an internal diameter of 2.4 mm for 1 min (10 mL/s). After centrifugation at 2000 × *g*/min for 3 min, the emulsion was divided into three layers: a top oil layer, middle ECM/SVF-gel layer, and bottom layer with a small amount of fluid. For SVF preparation, the Coleman adipose tissue was minced and incubated with 0.075% collagenase I (Sigma-Aldrich, St. Louis, MO, USA) at 37°C for 40 min. After terminating the digestion, the cell suspension was filtered through a 100 *μ*m mesh cell strainer, centrifuged at 500 × *g* for 3 min, and the cell pellet was resuspended in the Dulbecco's modified Eagle's medium (DMEM; Gibco, Carlsbad, CA, USA). ECM/SVF-gel and SVF suspension containing the same numbers of cells (5 × 10^5^) were cultured in 5 mL DMEM supplemented with 10% fetal bovine serum (FBS; Gibco) and 1% penicillin-streptomycin (Gibco) at 37°C with 5% CO_2_ for 24 h. The medium was replaced with serum-free medium to obtain CM. After a further 24 h, the supernatant was collected from the ECM/SVF and SVF cultures and filtered through a syringe filter unit (40 mm) to remove cell and tissue debris. The aliquots were stored at −20°C until use (Figure 1).

### 2.3. Growth Factor Assays

VEGF, bFGF, PDGF, and KGF in the ECM/SVF and SVF CMs were assayed using a Quantikine enzyme-linked immunosorbent assay kit (Sigma-Aldrich) according to the manufacturer's instructions.

### 2.4. Hair Growth Activity *In Vivo*

Seven-week-old C57BL/6 mice were anesthetized by intraperitoneal injection of 10 g/L pentobarbital sodium (0.4 mL/100 g), and their back hair was shaved with clippers and then completely removed using hair remover cream (Reckitt Benckiser France, CEDEX, France). Phosphate-buffered saline (PBS; control), ECM/SVF-CM, and SVF-CM, respectively, were then injected subcutaneously in the dorsal skin of each mouse once per week for 3 weeks to examine the hair-growth activities of the CMs. Skin darkening was monitored, and the hair-growth score was evaluated according to the method described by Vegesna et al. [22]. Briefly, a score of 0 indicated no change in hair growth compared with the hair-loss induction day, and a score of 10 indicated full hair growth over the entire site where the hair had been removed. Each group consisted of 10 mice.

### 2.5. Isolation, Culture, and Immunofluorescence Identification of Human DPCs

Normal human scalp hair follicles were obtained during hair transplantation procedures, and all follicles were in the anagen phase. DPCs were isolated and expanded as described previously [23]. Briefly, follicle bulbs were transected and dermal papillae (DP) were microdissected from the bulbs and transferred to cell culture in DMEM supplemented with 10% (*v*/*v*) FBS and 1% (*v*/*v*) penicillin-streptomycin. After 1 week of culture at 37°C with 5% CO_2_, the DPCs were treated with 0.25% (wt/vol) trypsin-EDTA (Gibco) and split into two equal volumes for subculture. DPCs at passage 3 were used in subsequent experiments.

Immunofluorescence staining was used to identify the characteristics of the DPCs, and alkaline phosphatase (ALP) and *β*-catenin were examined as molecular markers of DPCs. For immunofluorescence staining, cultured DPCs were fixed with 4% paraformaldehyde (Gibco) for 30 min at 4°C, rinsed with PBS (Gibco), and permeabilized with 5% Triton X-100 (Sigma) for 10 min, followed by blocking with 5% bovine serum albumin (BSA; Gibco) for 30 min. The DPCs were then stained with the following primary antibodies at 4°C overnight: rabbit anti-*β*-catenin (1 : 200, Abcam, Cambridge, UK) and rabbit anti-ALP (1 : 100, Abcam). After thorough washing with PBS, the cells were stained with Alexa Fluor488-conjugated goat anti-rabbit immunoglobulin G secondary antibody (1 : 200, Abcam) and DAPI (1 : 500, Sigma) at room temperature for 1 h. Immunofluorescent images were recorded using a fluorescence microscopy system (IX71 FL, Olympus, Japan).

### 2.6. DPC Proliferation Assay

DPCs at passage 3 were seeded into 96-well culture plates at a density of 4000 cells per well and cultured in DMEM supplemented with 10% FBS incubated at 37°C with 5% CO_2_ for 6 h to permit cell adhesion, to study the effects of the CMs on cell proliferation. The cells were then washed repeatedly with PBS and cultured in each of the two CMs or in serum-free medium (control group) for 1, 3, or 5 days, respectively. DPC proliferation was determined by CCK8 assay (Sigma). The absorbance of the culture medium was measured at 450 nm using a multilabel counter (*n* = 3).

### 2.7. Proliferative Activity of Cells in the Bulge Region

Half of the mice in each group were sacrificed 2 weeks after injection. The dorsal skin was harvested and fixed in 4% paraformaldehyde (Sigma) for at least 24 h, embedded in paraffin, and 4 *μ*m sections were cut. The proliferative activity of the cells in the bulge region of the hair follicle was examined in deparaffinized sections stained with anti-Ki-67 (1 : 100, Abcam). Specimens were pretreated by heating followed by blocking with 1% BSA (Sigma) and incubation with primary antibody at 4°C overnight. The sections were then incubated with secondary antibody (1 : 250, Abcam) for 30 min, stained with diaminobenzidine (Invitrogen) and hematoxylin, dehydrated, transparentized, and mounted. Ki67-positive cells of serial sections of specimens were counted with a microscope at high magnification. Statistical analysis was performed based on five high-powered fields per sample.

### 2.8. Angiogenesis Activity *In Vivo*

We determined the effects of ECM/SVF-CM and SVF-CM on angiogenesis *in vivo* by examining the vascularization of the hair-regeneration sites 2 weeks after the injection of CM. Angiogenesis levels were evaluated on the basis of gross observation of the inner skin of the hair-regrowth site and immunohistochemistry staining for CD31. Immunohistochemistry staining for CD31 was carried out as above, using anti-CD31 (1 : 200, Abcam). Neovascularization of serial sections of specimens was counted with a microscope at high magnification.

### 2.9. Western Blot Protein Assay

Protein samples were prepared in RIPA lysis buffer (Gibco), and 30 *μ*g aliquots were separated by sodium dodecyl sulfate-polyacrylamide gel electrophoresis and blotted onto polyvinylidene fluoride membranes. After blocking, the membranes were incubated with rabbit anti-Wnt5a (1 : 1000, Abcam), rabbit anti-Wnt10b (1 : 1000, Abcam), and anti-glyceraldehyde 3-phosphate dehydrogenase (1 : 1000, Abcam) monoclonal control antibody at 4°C for 24 h. Membrane-bound primary antibodies were detected by incubation with secondary antibodies (1 : 5000, Abcam) at room temperature for 1 h. The results were analyzed using an enhanced chemiluminescence kit (Invitrogen, Grand Island, NY, USA) and analyst/PC densitometry software (Bio-Rad Laboratories, Hercules, CA, USA).

### 2.10. Statistical Analysis

Differences among treatments were assessed by one-way analysis of variance (ANOVA) followed by Tukey–Kramer's test for independent groups. A *P* value < 0.05 was regarded as significant.

## 3. Results

### 3.1. Growth Factor Levels Increased in CM from ECM/SVF

**Figure 2:**
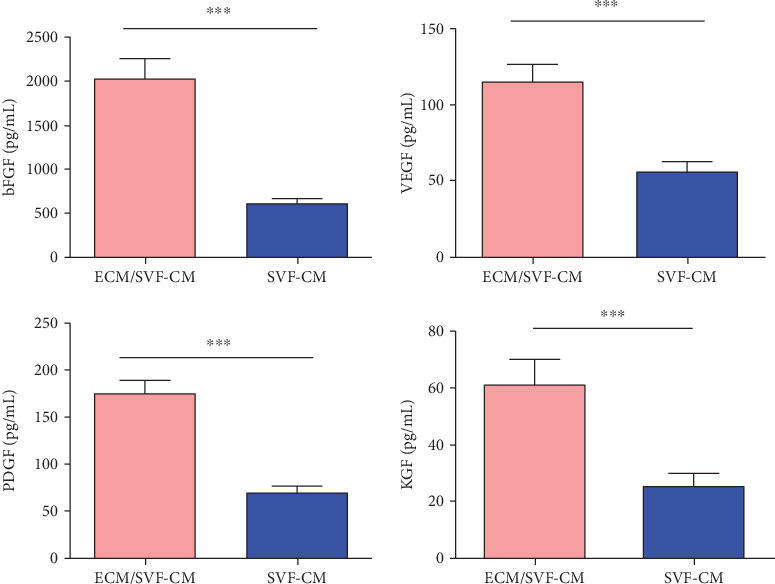
Quantification of growth factor expression. Quantification of VEGF, bFGF, PDGF, and KGF expression in SVF-CM and ECM/SVF-CM. ^∗∗∗^*P* < 0.01.

We investigated the regulatory factors in ECM/SVF and SVF CMs responsible for stimulating mouse hair growth. Levels of VEGF, bFGF, PDGF, and KGF were all significantly higher in ECM/SVF-CM than in SVF-CM (Figure 2).

### 3.2. Hair-Growth-Promoting Effects of ECM/SVF-CM in a C57BL/6 Mouse Model

**Figure 3:**
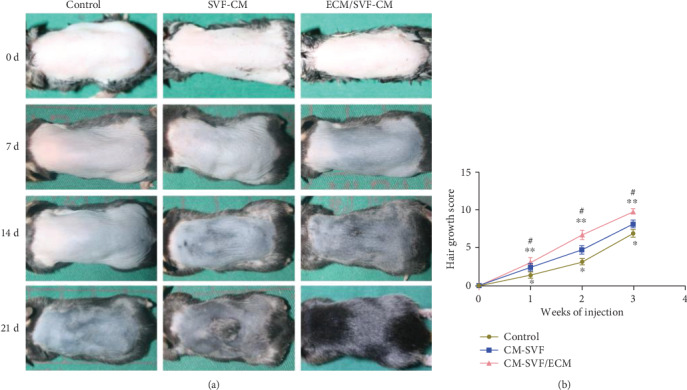
Hair-growth effect of ECM/SVF-CM in hair-loss-induced C57BL/6 mice. The hair was removed from the backs of C57BL/6 mice and the hair-growth rate was monitored for 3 weeks. Mice were injected with ECM/SVF-CM, SVF-CM, and PBS (control), respectively, once per week for 3 weeks. Gross view was observed by photographs (a), and hair-growth scoring was measured (b). Mice in the ECM/SVF-CM injection group (*n* = 10) showed significantly increased hair regeneration compared with the SVF-CM injection group (^∗∗^*P* < 0.05, *n* = 10) and negative control group (^#^*P* < 0.01, *n* = 10). The SVF-CM injection group also showed significantly increased hair regeneration compared with the control group (^∗^*P* < 0.05). The data shown represent one of each group, and experiments were performed three times independently.

We compared the differences in hair growth after weekly injection of ECM/SVF-CM and SVF-CM to determine their effects on hair growth efficiency in C57BL/6 mice. CM-injected mice showed enhanced hair-growth rates at 1 and 2 weeks after injection, compared with the negative control group injected with PBS. Moreover, mice injected with ECM/SVF-CM had more rapid hair growth than mice injected with SVF-CM (Figures 3(a) and 3(b)). The ECM/SVF-CM group developed dark pigment on the initially pink hairless skin 1 week after injection, while the skin was less dark in the SVF-CM group and remained pink in the negative control group (Figure 3(a)). Hair recovery extent on the dorsal skin and hair length were both significantly affected in the ECM/SVF-CM injection group at 2 weeks after injection (Figure 3(a)), and 95%–100% hair regeneration to full length was observed in the ECM/SVF-CM group, but not in the SVF-CM group (70%–75%) at 3 weeks after injection. These results suggest that both CMs enhanced hair growth and that CM from ECM/SVF had a stronger ability to simulate hair growth.

### 3.3. ECM/SVF-CM Increased Proliferation of Human DPCs

**Figure 4:**
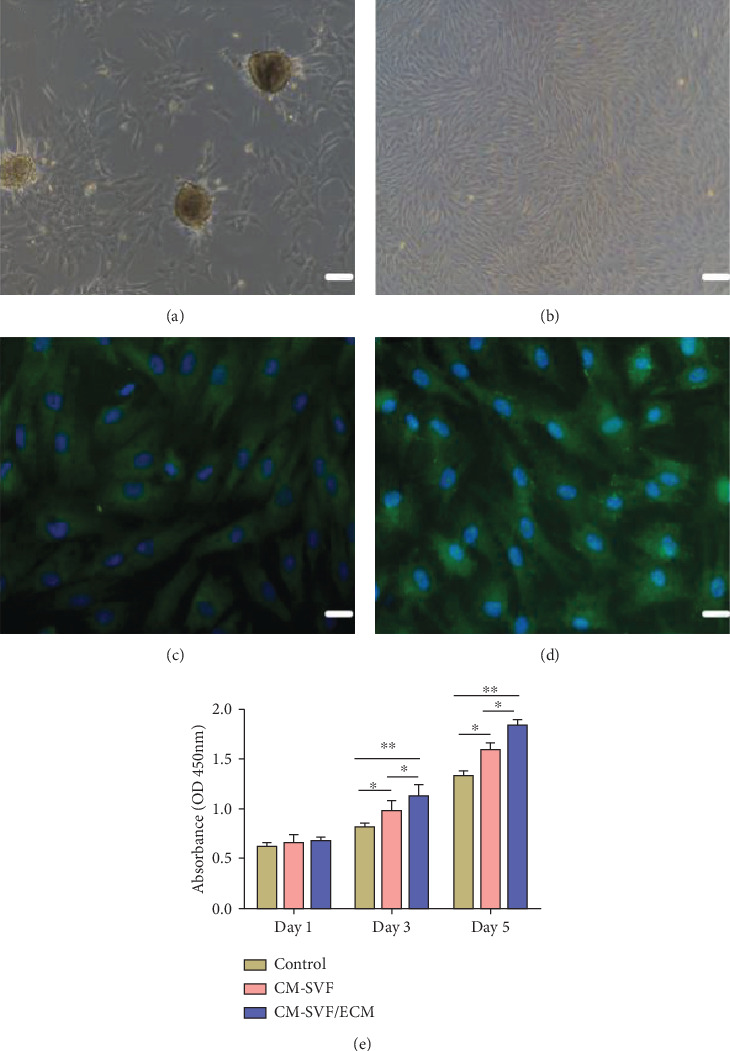
Morphology of DPs and DPCs. Immunofluorescence identification of DPCs and proliferation-promoting effect of the two CMs on DPCs. Phase-contrast micrograph showing oval appearance 2 days after inoculation, with most DPs attached with a few DPCs around them (a). DPCs showing spindle-shaped morphology (b). ALP (c) and *β*-catenin (d) were highly expressed in DPCs as shown by immunofluorescence staining. The viabilities of DPCs in the CM-treated groups were significantly higher than in the control group at days 3 and 5, and the viability in the ECM/SVF-CM-treated group was significantly higher than in the SVF-CM-treated group (e). Scale bar = 100 *μ*m. (^∗^*P* < 0.05, ^∗∗^*P* < 0.01).

Hair follicle regeneration is governed by the interaction between epithelial precursor cells residing in the bulge region and mesenchymal cells located at the base of the hair follicle, known as the DP [24]. We investigated the effects of CMs on DPC proliferation. DPCs were isolated, cultured, and subjected to immunofluorescence analysis. The DPs were oval and most dermal DPs were attached with a few DPCs around them at 2 days after incubation (Figure 4(a)). DPCs proliferated in an adherent fashion with spindle-like morphology (Figure 4(b)). To identify the intrinsic characteristics of DPCs, signature markers associated with the hair-inducing ability of DPCs, including ALP and *β*-catenin, were identified by immunofluorescence staining. ALP and *β*-catenin were highly expressed in DPCs (Figures 4(c) and 4(d)). The effects of the CMs on DPC proliferation are shown in Figure 4(e). The relative viabilities of DPCs in the CM-treated groups were significantly higher than in the control group at days 3 and 5, while the viability of the ECM/SVF-CM-treated cells was significantly higher than that of the SVF-CM-treated cells.

### 3.4. ECM/SVF-CM Increased Proliferation of Cells in the Bulge Region of Hair Follicles

**Figure 5:**
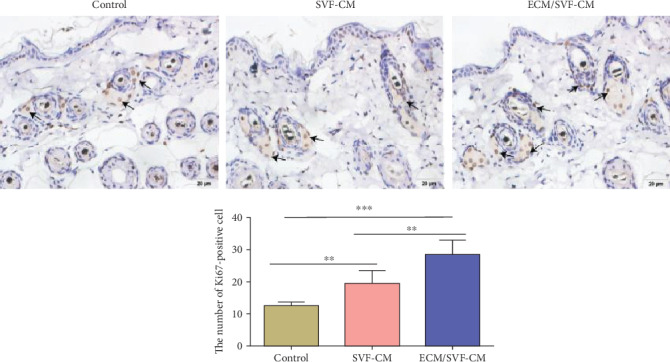
Immunohistochemical staining of Ki-67 in bulge cells. The number of Ki-67-positivity cell was significantly more in both the ECM/SVF-CM- and SVF-CM-treated groups compared with the control group, with the highest cell viability in the ECM/SVF-CM-treated group (^∗∗^*P* < 0.05, ^∗∗∗^*P* < 0.01). Scale bar = 20 *μ*m.

Previous studies revealed the existence of a pool of hair follicle stem cells in the bulge region [25]. These cells provide abundant progeny that are responsible for sustaining the intact structure of the hair follicle during continuous hair cycles [25]. We evaluated Ki-67 expression in the bulge cells by immunohistochemical staining to determine the effects of the CMs on cell proliferation in this region of the hair follicle. Cell proliferation rates in both the ECM/SVF-CM- and SVF-CM-treated groups were higher than those in the control group, with the highest cell viability in the ECM/SVF-CM-treated group (Figure 5).

### 3.5. ECM/SVF-CM Increased Neoangiogenesis in C57BL/6 Mice

**Figure 6:**
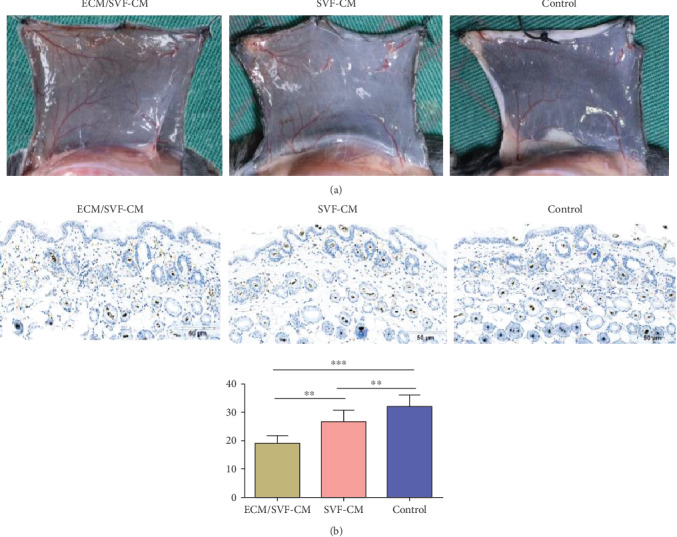
Stimulatory effect of ECM/SVF-CM on angiogenesis. Vascularization of the hair-regeneration sites was analyzed 2 weeks after injection. The inner skin of the hair-regrowth site showed mature blood vessel branching (a). Among the three groups, the ECM/SVF-CM-injection group showed markedly enhanced formation of mature vessels (a). Immunohistochemical staining of CD31 showed more new blood vessels in the ECM/SVF-CM-injection compared with the SVF-CM-injection and control groups. Arrow indicates blood vessels (b). (^∗∗^*P* < 0.05, ^∗∗∗^*P* < 0.01). Scale bar = 50 *μ*m.

We investigated vascularization of the hair-regeneration sites 2 weeks after injection, to determine the effects of the CMs on angiogenesis *in vivo*. Blood vessels in the inner dorsal skin in the CM-treated groups were increased compared with the control group (Figure 6(a)). Mice in the ECM/SVF-CM-treated group showed clear blood vessel branching and a significantly enhanced level of mature vessel formation (Figure 6(a)). We confirmed this by immunohistochemistry analysis of CD31. As expected, CD31 expression levels were significantly higher in the ECM/SVF-CM-treated compared with the SVF-CM-treated and control groups (Figure 6(b)).

### 3.6. Analysis of Wnt5a and Wnt10b Expression Involvement in Hair-Growth Promotion by ECM/SVF-CM

**Figure 7:**
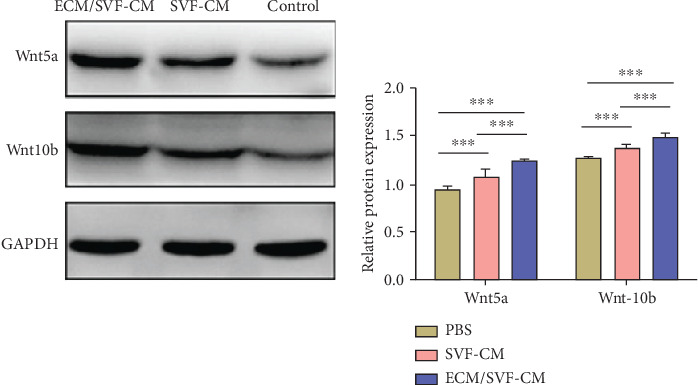
Involvement of Wnt pathway in the stimulatory effect of hair growth by ECM/SVF-CM. Both ECM/SVF-CM and SVF-CM markedly increased Wnt5a and Wnt10b expression levels compared with the control group, with the highest expression in the ECM/SVF-CM-treated group (^∗∗∗^*P* < 0.05).

To investigate the signaling mechanism underlying the induction of anagen phase in the CM groups, we detected signature proteins associated with anagen induction by western blot. Mice treated with CMs exhibited anagen induction, as evidenced by high expression of Wnt5a and Wnt10b (Figure 7). In addition, the ECM/SVF-CM-treated group had significantly higher Wnt5a and Wnt10b expression levels compared with the group treated with SVF-CM. These results suggest that CMs activate certain functions in the anagen phase, and that CMs stimulate hair follicle growth through the Wnt pathway.

## 4. Discussion

To optimize the CM therapy strategy for stimulating hair growth, we introduced a novel adipose tissue-derived stem cell and adipose native ECM enrichment product (ECM/SVF-gel) and compared the effects of ECM/SVF-CM and SVF-CM on hair growth. The results suggested that ECM/SVF-CM exerted hair growth-promoting effects via the proliferation of DPCs and cells in the bulge, and by neovascularization and anagen induction.

Hair follicles undergo cyclic transformation from rapid growth (anagen) to regression (catagen) and then progress to relative quiescence (telogen). The hair-growth cycle is fine-regulated by epithelial–dermal interactions [26]. In anagen, hair growth is governed by the proliferation of follicular cells, mainly composed of epithelial cells residing in the bulge and DPCs located at the base of the hair follicle [26]. In addition, hair growth can be induced by modulating the hair cycle, for example by delayed catagen induction and prolonged anagen, or by transition from telogen to anagen [27, 28]. Localized supplementation of growth factors and cytokines favoring hair growth was reported to promote hair regeneration [8]. Previous reports show that ADSC-CM promoted hair growth by regulating the cell cycle and inducing the anagen phase of the hair cycle, thereby promoting the proliferation of DPCs and possibly epithelial cells [9]. Our current results are consistent with those findings.

The paracrine effect is one of the most important therapeutic advantages of stem cell therapy [9]. Secretory factors derived from ADSCs related to hair-growth stimulation, including VEGF, bFGF, PDGF, HGF, KGF, and IGF, positively regulate hair follicle activity and promote hair growth [8]. The population of SVF includes endothelial cells, endothelial progenitor cells, pericytes, preadipocytes, macrophage, and ADSCs [29]. However, cells were almost excluded except for ADSCs after *in vitro* cell culture. In our previous studies, we found that the percentage of MSC-specific cell markers (CD90^+^, CD73^+^, or CD105^+^) in cultured SVF cells were more than 95%, while the percentage of hematopoietic-specific cell markers (CD34^−^, CD11b^−^, CD19^−^, or CD45^−^) was negative [30–32]. In this study, CMs were collected from the medium of ECM/SVF and SVF after cultured for 48 h. Therefore, we consider that the biological efficacy of CMs mainly attributed to ADSCs. The expression of growth factors (bFGF, VEGF, PDGF, and KGF) was found in ECM/SVF-CM and SVF-CM which may play important roles in regulating cell functions during hair growth. In particular, bFGF promotes hair growth via DPC proliferation and increased hair follicle size [33] and also has a positive effect on the hair growth cycle by inducing anagen phase and elongating the hair shaft [34]. PDGF induces and maintains anagen phase and provides a stem cell niche to regulate the hair cycle [35]. KGF prevents hair follicle cell death by promoting protein synthesis [36], and HGF contributes to anagen maintenance by retarding hair follicle regression and suppressing follicle keratinocyte apoptosis [37]. Enhancement of angiogenesis is usually accompanied by positive hair growth promotion [38]. VEGF is a powerful mediator of angiogenesis that accelerates hair growth and increases follicular size by initiating angiogenesis, resulting in the formation of a new network of capillaries carrying blood, oxygen, and nutrients to the hair follicle [39]. Angiogenesis around hair follicles was previously shown to be related to the hair cycle [40]. Significant angiogenesis occurs during the hair-growth anagen phase, while vessels around the hair follicles are reduced and degenerate during the hair growth-retardation catagen and telogen phases [41]. In the current study, the hair-growth effect of CM from ECM/SVF was associated with a stimulatory effect on angiogenesis, as evidenced by a marked increase in vessels following treatment. Inducing angiogenesis may also induce the anagen phase of the hair cycle as a hair growth-stimulating process. Therefore, we speculated that the promotion of hair growth in the two CM-treatment groups may be due to the growth factors in the extract.

Optimization of stem cell-CM to upregulate key growth factors is an important strategy to achieve encouraging outcomes in terms of hair regeneration and hair growth. The current study showed that VEGF, bFGF, PDGF, and KGF secretions were all significantly increased in ECM/SVF-CM compared with SVF-CM, resulting in more effective stimulation of hair growth. This result may be attributed to the beneficial effects of native ECM on tissue-resident stem cells. Natural ECM provides a physical scaffold for cells and retains the natural state of the cells, thereby providing a favorable environment for attached cells to maintain optimal cell survival and efficacy [21]. In addition to providing structural support for cells, ECM also acts as a dynamic microenvironment, regulating important biological functions of cells [21]. In this study, we found that ECM/SVF-CM had a higher concentration of growth factors than the SVF-CM, indicating a higher cell potency in ECM/SVF-gel. However, further research is still necessary to verify the precise mechanisms underlying the higher expression of growth factors in ECM/SVF-CM compared with SVF-CM.

Although we found the beneficial effect of ECM/SVF-CM on hair growth, some related limitations still exist. First, we did not compare ECM/SVF with an *in vitro* analogue. Second, the components of CMs were complex, and mass spectrometry analysis of the components stimulated by ECM/SVF-CM would be necessary. Furthermore, we did not explain the interaction between cells and ECM.

## 5. Conclusions

In conclusion, this study provides the first evidence for the promoting effects of CM from ECM/SVF on hair growth in a mouse model, acting via the proliferation of DPCs and cells in the bulge, as well as by enhancing angiogenesis and anagen induction. ECM/SVF-CM can be stored with long-term stability and can be produced on a large scale, making it a potentially low-cost therapy. Further studies are needed to confirm the use of CM from ECM/SVF as an effective and improved strategy for the treatment of hair loss.

## Data Availability

The data used to support the findings of this study are included within the article.
